# Health-related quality of life (HRQOL) and its associated factors in children with Type 1 Diabetes Mellitus (T1DM)

**DOI:** 10.1186/s12887-017-0788-x

**Published:** 2017-01-13

**Authors:** Marta Murillo, Joan Bel, Jacobo Pérez, Raquel Corripio, Gemma Carreras, Xavier Herrero, Josep-Maria Mengibar, Dolors Rodriguez-Arjona, Ulrike Ravens-Sieberer, Hein Raat, Luis Rajmil

**Affiliations:** 1 Pediatric Service, University Hospital Germans Trias i Pujol, Ctra de Canyet s/n, Badalona 08916 Barcelona, Spain; 2 Autonomous University of Barcelona, Barcelona, Spain; 3 Department of Pediatric Endocrine, Hospital of Sabadell, Corporació Sanitària Parc Taulí, Parc Taulí s/n 08208 Sabadell, University Institute Parc Taulí—UAB, Autonomous University of Barcelona, Campus d’Excelència Internacional, Sabadell, Spain; 4 Pediatric Endocrinology, Hospital Sant Pau, Autonomous University of Barcelona, c/Sant Quintí 89, 08026 Barcelona, Spain; 5 Corporació de Salut del Maresme i la Selva, Sant Jaume, 209-217, 08370 Calella, Spain; 6 Catalan Agency for Health Quality and Assessment, Roc Boronat 81-95, 08005 Barcelona, Spain; 7 Department of Child and Adolescent Psychiatry, Psychotherapy, and Psychosomatics, University Medical Center Hamburg- Eppendorf, Martinistr, 52 (W29), 20246 Hamburg, Germany; 8 Department of Public Health, Erasmus MC – University Medical Center Rotterdam, 3075 EA Rotterdam, The Netherlands; 9 IMIM Hospital del Mar Institute of Medical Research, Dr Aiguader 88, 08003 Barcelona, Spain; 10 Centro de Investigación Epidemiológica en Red (CIBERESP), 28029 Madrid, Spain

**Keywords:** Adolescents, Children, Health-related quality of life, Pediatric, Type 1 diabetes

## Abstract

**Background:**

The objective of the study was to describe the baseline health-related quality of life (HRQOL) in a cohort of children and adolescents with type 1 diabetes mellitus (T1DM), and analyze its associated clinical and sociodemographic factors, assessing HRQOL through internet.

**Methods:**

This was a descriptive study of 136 patients with T1DM from 5 hospitals in Catalonia, Spain (72 girls, mean age 13.4 years (range 8–19). Inclusion criteria were more than 6 months from diagnosis, more than 8 years old and without cognitive problems. Sociodemographic (age, sex, family level of education, type of family and origin) and clinical variables (type of insulin therapy, duration of disease, adherence to treatment, body mass index and HbA1c) were collected. HRQOL was assessed using the EuroQol-5D (EQ-5D-Y) and KIDSCREEN, collected via web. Mental health status was assessed using the Strengths and Difficulties Questionnaire. Multiple linear regression models were adjusted.

**Results:**

Physical-well-being mean scores were lower (worse) than the European average (<50) and especially in girls, older children (>11 years old), those from single-parent families, and those with low adherence. Older children and patients with poor metabolic control (HbA1c >7,5% [58 mmol/mol]) showed worse scores in the KIDSCREEN-10 index. Similar results were observed with the EQ-5D-Y. Multivariate models showed that age, single-parent families, adherence and mental health were the most influential factors.

**Conclusions:**

Diabetic patients report similar HRQOL than the population of the same age with slightly worse physical well-being. The study shows some factors to be taken into account to improve HRQOL, and also the feasibility of using web to collect information in clinical practice.

## Background

There is a growing interest in the study of health-related quality of life (HRQOL), which has become an important end-point measure from the clinical and epidemiological point of view [1].

Type 1 Diabetes Mellitus (T1DM) is a chronic disease that affects all aspects of patient’s life and especially psychologically and therefore HRQOL. The management of T1DM is complex, requiring a high degree of responsibility and self-control to achieve an adequate metabolic control. Key aspects to succeed are the support of a multidisciplinary team, education in disease management with decision-making capacity, and exploit the possibilities offered by new technologies without forgetting the emotional sphere of the patient and family. In fact, treatment guidelines recommend routine screening for emotional status and family relationships [2]. On the other hand, hormonal and psychosocial changes that occur during puberty make this stage a difficult time with high metabolic instability, and many adolescents experience deterioration in metabolic control [3, 4].

Assessment of HRQOL in clinical practice is important in order to evaluate the course of the disease, early detection of problems, and to determine what type of insulin therapy would be adequate to maintain acceptable metabolic control with less impact on HRQOL in each patient [2, 4].

Previous studies have shown that girls, older children, and specific sociodemographic factors such as single-parenthood, lower family income or minority status would be associated with poor metabolic control and worse HRQOL [2]. Studies addressed to analyze emotional and psychosocial aspects such as self-management and self-esteem, resilience, and parenting showed a positive influence on HRQOL, although interventions need to be tailored to the specific needs of children and families [5]. A systematic review assessed HRQOL in children with T1DM [6] and its potential help in clinical management. This review describes HRQOL in groups of patients comparing with control groups, and also analyzing HRQOL with specific instruments. The results of this review show that overall diabetic children have similar HRQOL compared to healthy peers but reported disease specific problems. Gender and age differences were also found. Studies included in this review were mainly descriptive and no studies were included comparing with general population data.

A few studies were carried out in Spain addressing HRQOL in children with T1DM [7], Studies analyzing clinical factors associated with HRQOL in patients with T1DM, and on the use of internet to collect information [8] and its usefulness on clinical practice [9–11] were carried out in other contexts.

The aim of this study was to describe the HRQOL in Spanish children and adolescents with T1DM and analyze its associated clinical and sociodemographic factors, assessing HRQOL through internet. This study represents the baseline assessment of an intervention to evaluate use of generic HRQOL measures administered via internet in clinical practice. According to previous studies it was expected that children from vulnerable families, with poor metabolic control, and/or poor mental health would show worse HRQOL.

## Methods

### Participants and procedures

Patients were consecutively recruited from a list of 205 potential candidates (104 girls) between 8 and 19 years old with T1DM attending outpatient pediatric endocrinology of 5 hospitals in the Barcelona province area, Spain (7 pediatric endocrinologists participated in the study), between July and December 2014. Exclusion criteria were less than 6 months from diagnosis of T1DM, less than 8 years old at the time of recruitment, cognitive problems that prevented comprehension of the questionnaires, and patients who declined to participate in the study.

Families that fulfilled inclusion criteria were provided with a letter explaining the characteristics of the project and the need to access to digital questionnaires 48 h before the follow-up visits. After agreeing to participate in the study, parents answered a paper questionnaire administered during this visit including information on sociodemographics and family’s variables. Before the next quarterly routine visit, a reminder was sent by email and/or telephone to the family and patient.

### Study variables

Sociodemographic variables were collected from parents, clinical variables came from clinical records, and HRQOL, mental health and adherence to treatment came from the self-administered online questionnaire.

Sociodemographic variables were: age, sex, and the highest family level of education (primary, secondary or university). The variable family origin (native vs immigrant status) was based on the country where the child and parents were born. It was considered as immigrants if the child and/or both parents were born in a developing country (Asia, Africa, Latin America, or Eastern European country). Family type (single-parent vs bi-parental family), and hospital were also collected.

Clinical variables analyzed were: type of insulin therapy (multiple daily injections [MDI] or pump therapy, only in 2 cases); use of insulin bolus calculator or not; and time since diagnoses (in years). Decompensation in the last 3 months was assessed as significant hypoglycemia (<60 mg/dl with decreased level of consciousness requiring glucagon or the help of others to be reversed), and significant hyperglycemia (>400-450 mg/dl which required action by the professionals). Diabetes ketoacidosis was also collected. The HbA1c was used as a measure of metabolic control by determination in capillary blood (DCA 2000 Bayer/Siemens®) or serum in an analytical laboratory (HPLC). Weight, height and body mass index (BMI, Z score) [12] was also collected.

The web-version of the questionnaires was developed through a generic internet tool using Ruby on Rails applications and MySQL database (http://rubyonrails.org).

The self-administered questionnaire included the internet versions of the Euroqol-5D-Y (EQ-5D-Y) and KIDSCREEN-27 and KIDSCREEN-10 index as measures of HRQOL.

The self-reported EQ-5D-Y [13] descriptive system consists of 5 dimensions (mobility, self-care, usual activities, pain-discomfort and anxiety-depression) with 3-levels Likert response scales (no problems, moderate problems and serious problems) and a visual analogue scale (VAS) on the general health status from 0 (worst health status) to 100 (best health status possible). The Internet Catalan and Spanish versions of the eEQ-5D-Y have demonstrated acceptable reliability and validity in the Spanish population, and similar to the paper version [14].

The self-reported KIDSCREEN-27 [15] was assessed by its 5 dimensions: physical well-being (5 items), psychological well-being (7 items), autonomy and relationships with parents (7 items), social support and relationship with friends (4 items) and school environment (4 items). Responses were categorized into 5 options Likert scales that assess the frequency or intensity of the attribute, with a recall-period of 1 week in most questions. The scores are standardized to a mean of 50 and a standard deviation (SD) 10, from a reference sample of 22,000 European children and adolescents. The KIDSCREEN-10 index was also included as a summary measure. The internet Spanish and Catalan versions of the questionnaire have demonstrated acceptable reliability and validity in this population, similar to the paper version [16].

Children’s mental health status was assessed using the Strengths and Difficulties Questionnaire (SDQ), a brief behavioral screening questionnaire for children and adolescents that asks about their mental health symptoms and positive attitudes [17]. The instrument consists of 25 items measuring 5 dimensions. All items are scored on a three point scale (0 = not true, 1 = somewhat true, and 2 = certainly true). Items in the 4 problem dimensions are summed to give a total difficulties score ranging from 0 (no problems) – 40 (maximum problems). Higher scores indicate more problems. The Spanish and catalan versions have been shown to be reliable and valid [18].

Adherence to treatment in the month prior to the interview was assessed by two questions: How often have you checked your blood sugar?; and: How often did you do a blood sugar check within 2–3 h after a meal? Both questions included 7 and 5 frequency Likert-scale answer categories, respectively. Patients were categorized into high adherence if they answered a frequency of control of glucose level 3 or more times per day, and also a frequency of blood sugar check within 2–3 h after a meal at least 3 or more times a week.

### Statistical analysis

Mean scores of the KIDSCREEN-27 dimensions and KIDSCREEN-10 index and its 95% confidence interval (95% CI) were computed and compared with the average of European population. The distribution of health states were also analyzed according to the descriptive system of EQ-5D-Y. Mean scores of HRQOL, both the KIDSCREEN and VAS score, according to sociodemographic factors, adherence and control of diabetes, and clinical variables were assessed by Student *t* test, Mann–Whitney U, or ANOVA according to the characteristics of analyzed variables. Standardized mean differences (effect size, ES) estimated as the difference between the means divided by pooled standard deviation, were computed to analyze the magnitude of differences [19].

Effect sizes of 0.2–0.5 were considered small; those between 0.51 and 0.8 moderate, and those over 0.8 were considered large.

Multiple linear regression models were adjusted to analyze the influence of factors associated with HRQOL controlling for socio-demographic, clinical factors, and mental health. Interactions terms between clinical and sociodemographic factors were also explored. Results of regressions in terms of B coefficients can be interpreted as a modification (increase [+] or decrease [−]) on the dependent variable (a given dimension of HRQOL) for every unit of change in the predictor variable (e.g. age). Bonferroni correction was used to control for multiple comparisons. Program STATA.11 software versions were used in the analysis.

## Results

One hundred thirty six patients were included in the study (participation rate 65.5%). Sixty one patients were not included due to change of address, transfer to the adult unit, or not attending follow-up visits, while 8 patients rejected their participation in the study. There were no significant differences in terms of age, gender or years of disease progression among patients who entered in the study and those not included.

Table 1 shows the clinical and demographic characteristics of the sample. The average age of participants was 13.4 years; 52.9% were girls; 15.7% came from single-parent families; 6% of immigrant families, and 19.4% of parents had university degree. In 4 cases the questionnaires were filled out in a waiting time previous to the visit given the absence of internet at home.

**Table 1:** Sociodemographic and clinic characteristics of participants

Sociodemographic variables	N	Mean (SD) or %
Age		
Mean	136	13.45 (2.9)
8–11y	36	26.5
12–19y	100	73.5
Sex		
Boys	64	41.1
Girls	72	52.9
Type of family		
Biparental	113	84.3
Monoparental	21	15.7
Highest family level of education		
Primary school	54	40.3
Secondary school	54	40.3
University degree	26	19.4
Origin		
Native	128	94.1
Immigrant	8	6.0
Clinical variables		
Time w/diagnoses (years)	136	5.04 (3.73)
> 5 years	54	39.7
≤ 5 years	82	60.3
BMI (Z score)	136	0.24 (0.9)
HbA1c	136	7.65 (1.34)/60 mmol/mol (14.6)
> 7.5% (58 mmol/mol)	65	47.8
≤ 7.5%	71	52.2
Mental health		
SDQ	136	10.64 (5.28)
Adherence		
High	64	47.1
Low	72	52.9
Changes during the last 3 months		
No changes on treatment	110	81.5
Changes on diet/nutrition	18	13.3
Bolus calculator	7	5.2
Hypoglycemia		
Yes	3	2.2
No	133	97.8
Hyperglycemia		
Yes	10	7.4
No	126	92.6

Missing values: level of education (2); type of family (2); changes during the last 3 months (1)

*SD* Standard deviation, *BMI* body mass index, *HBAc1* Glycated hemoglobin, *DSMQ* Diabetes Self Management Questionnaire

The mean time of disease progression was 5 years, with 39.7% (*n =* 54) longstanding (>5 years), and 19.9% less than 1 year. The average HbA1c level was 7.65% (SD ± 1.3; 60 mmol/mol; SD ± 13.2); 52.2% of patients had good metabolic control (HbA1c <7.5%; 58 mmol/ml), with an average of 8.64% (SD ±1.22; 71 mmol/ml) in those with poor metabolic control and 6.74% (SD ± 0.59; 50 mmol/ml) in those with good metabolic control; 1.5% of patients used pump therapy, the rest received MDI therapy; 81.5% of patients had no significant change in insulin therapy during the 3 months prior to baseline assessment. Five percent started to use an insulin bolus calculator, and 52.9% had a high level of adherence to treatment. There were 3 cases of significant hypoglycemia in the last three months. Mean score on mental health (SDQ total difficulties score) was 10.65.

The results of the KIDSCREEN-27 and the KIDSCREEN-10 mean scores, and VAS are shown in Table 2. Physical-well-being scores were lower than the European average (<50) and especially in girls, older children (>11y), those from single-parent families, and those with low adherence to the treatment. Older children (>11y = 48.3, [95% CI] 46.7–49.9), and patients with poor metabolic control (48.0; 95% CI 46.3–49.6) presented worse scores in the KIDSCREEN-10 index. On the other hand, patients with good metabolic control and shorter duration of disease (<5 years), showed better scores. Similar results were observed with the VAS. Table 3 shows the magnitude of differences (ES) on HRQOL according to sociodemographic and clinical variables. Large ES were seen on age, changes on treatment during the last 3 months, hyperglycemia and hypoglycemia.

**Table 2:** KIDSCREEN-27, KIDSCREEN-10 Index and Visual analogue scale (VAS) scores and its 95% confidence interval (95% CI) according to sociodemographic and clinical variables

	Physical well being	Psychological well-being	Parents/Autonomy	Peers	School	KIDSCREEN-10 index	VAS
Total	46.4 (44.8–48.0)	49.7 (48.0–51.3)	50.5 (49.1–51.9)	53.6 (52.1–55.2)	52.0 (50.4–53.6)	49.6 (42.2–51.0)	80.2 (77.6–82.7)
Age							
8–11y	49.2 (46.3–52.0)	52.5 (49.7–55.4)	50.9 (48.3–53.5)	55.6 (52.7–58.5)	56.1 (53.3–58.9)	53.3 (50.6–56.1)	85.9 (82.3–89.5)
12–19y	45.4 (43.5–47.3)	48.6 (46.7–50.6)	50.4 (48.8–52.1)	52.9 (51.1–54.8)	50.6 (48.7–52.4)	48.3 (46.7–49.9)	78.1 (75.1–81.2)
Sex							
Boys	47.4 (45.3–49.5)	50.0 (48.1–51.9)	50.2 (48.3–52.0)	54.3 (52.1–56.4)	49.7(47.8–51.7)	49.6 (47.9–51.2)	80.0 (76.4–83.5)
Girls	45.6 (43.2–48.0)	49.4 (46.7–52.0)	50.9 (48.8–52.9)	53.1 (50.8–55.3)	54.0 (51.6–56.4)	49.7 (47.4–51.9)	80.3 (76.8–83.9)
Type of family							
Biparental	47.4 (45.6–49.1)	50.1 (48.3–51.8)	51.0 (49.5–52.5)	54.0 (52.3–55.7)	52.3 (50.6–54.1)	49.9 (48.4–51.5)	81.4 (78.7–84.0)
Monoparental	41.5 (38.2–44.1)	47.9 (42.6–53.3)	47.9 (43.9–51.9)	50.5 (46.4–50.7)	50.0 (45.9–54.1)	47.4 (43.9–51.0)	75.5 (69.2–81.9)
Highest family level of education							
Primary school	45.2 (42.4–48.1)	49.0 (46.3–51.8)	49.5 (47.5–51.6)	52.8 (50.3–55.3)	52.0 (49.4–54.5)	49.2 (46.8–51.6)	80.2 (76.2–84.1)
Secondary school	46.7 (44.2–49.3)	50.2 (47.3–53.1)	51.6 (49.1–54.1)	53.3 (50.8–55.9)	52.0 (49.5–54.4)	50.0 (47.9–52.1)	79.5 (75.3–83.8)
University degree	47.9 (45.2–50.5)	50.1 (47.1–53.1)	50.2 (47.3–53.2)	55.0 (51.7–58.3)	51.9 (48.1–55.7)	49.3 (45.9–52.7)	83.0 (78.9–87.0)
Changes during the last 3 months							
No changes on treatment	47.2 (45.4–48.9)	50.4 (48.5–52.3)	50.8 (49.2–52.4)	53.9 (52.2–55.6)	52.2 (50.4–53.9)	50.2 (48.7–51.7)	81.6 (78.9–84.2)
Changes on diet/nutrition	42.4 (37.8–46–9)	45.7 (42.2–49.3)	50.1 (46.5–53.8)	51.6 (47.2–61.6)	50.6 (46.6–54.7)	46.7 (42.6–50.9)	71.7 (63.7–79.6)
Bolus calculator	42.2 (36.0–48.3)	46.4 (39.6–53.1)	47.1 (40.9–53.4)	52.1 (43.2–61.1)	51.0 (43.2–58.8)	46.1 (38.1–54.1)	77.2 (78.0–86.4)
Hypoglycemia							
Yes	51.8 (40.3–63.2)	60.5 (53.1–68.0)	49.2 (45.2–53.1)	57.9 (48.5–67.4)	65.5 (60.1–70.9)	59.9 (52.0–67.7)	90.6 (86.0–95.2)
No	46.3 (44.6–47.9)	49.4 (47.7–51.1)	50.6 (49.1–52.0)	53.5 (51.9–55.1)	51.7 (50.1–53.3)	49.4 (48.0–50.8)	79.9 (77.4–82.5)
Hyperglycemia							
Yes	41.9 (35.3–48.4)	45.4 (37.9–52.9)	50.2 (46.3–54.2)	50.0 (42.7–57.4)	50.7 (44.1–57.4)	47.2 (41.8–52.7)	70.2 (58.9–81.4)
No	46.8 (45.1–48.4)	50.0 (48.3–51.7)	50.6 (49.1–52.1)	53.9 (52.3–55.5)	52.1 (50.4–53.8)	49.8 (48.3–51.3)	81.0 (78.4–83.5)
HbAc1							
> 7.5% (58 mmol/mol)	44.3 (42.1–46.9)	48.4 (46.0–50.8)	49.7 (47.9–51.5)	51.9 (48.9–53.2)	51.1 (48.9–53.2)	48.0 (46.3–49.6)	77.3 (73.4–81.1)
≤7.5% (58 mmol/mol)	48.3 (46.1–50.6)	50.8 (48.5–53.2)	51.3 (49.2–53.4)	55.2 (53.1–57.4)	52.9 (50.6–55.3)	51.2 (48.9–53.4)	82.9 (79.7–86.0)
Time w/diagnoses							
≤5 years	47.5 (45.4–49.5)	50.6 (48.6–52.6)	51.0 (49.4–52.6)	54.8 (52.9–56.8)	52.9 (50.8–54.9)	50.7 (48.8–52.5)	79.9 (76.9–83.0)
> 5 years	44.8 (42.2–47.4)	48.2 (45.3–51.1)	49.8 (47.2–52.4)	51.8 (49.2–54.4)	50.8 (48.3–53.3)	48.0 (45.8–50.2)	80.5 (76.1–84.9)
Adherence							
High	48.6 (46.6–50.6)	51.2 (48.7–53.1)	50.4 (48.5–52.6)	54.0 (51.6–56.5)	52.6 (50.1–55.1)	51.1 (48.9–53.3)	82.7 (79.5–85.8)
Low	43.9 (41.8–46.1)	48.2 (46.1–50.3)	50.5 (48.5–52.3)	53.1 (51.2–55.0)	51.4 (49.4–53.4)	48.3 (46.6–50.1)	78.2 (74.4–82.4)

**Table 3:** Standardized mean differences (effect size, ES) according to sociodemographic and clinical variables

	Physical well being	Psychological well-being	Parents/Autonomy	Peers	School	KIDSCREEN-10 index	VAS
Age							
8–11y^a^	0.34	0.39	0.04	0.26	**0.58**	**0.61**	**0.56**
12–19y							
Sex							
Boys	–0.27	−0.05	0.10	−0.10	**0.48**	0.005	0.0001
Girls^a^							
Type of family							
Biparental^a^	**0.67**	0.21	0.26	0.37	0.24	0.30	0.42
Monoparental							
Primary school	0.29	0.13	0.10	0.25	0.03	0.01	0.21
Secondary school	0.16	0.002	0.14	0.20	0.06	0.09	0.21
University degree^a^							
Changes during the last 3 months							
No changes on treatment^a^							
Changes on diet/nutrition	**0.54**	0.48	0.06	0.24	0.15	0.43	**0.70**
Bolus calculator	0.54	0.40	0.43	0.18	0.12	0.51	0.32
Hypoglycemia							
Yes^a^	0.37	1.16	−0.16	0.49	1.52	**1.28**	−0.34
No							
Hyperglycemia							
Yes^a^	**−0.61**	−0.47	−0.03	−0.41	−0.14	−0.31	**−1.02**
No							
HbAc1							
> 7.5% (58 mmol/mol)^a^	**−0.5**	−0.23	−0.17	−0.33	−0.18	−0.38	−0.40
≤7.5% (58 mmol/mol)							
Time w/diagnoses							
≤5 years^a^	0.23	0.24	0.14	0.32	0.21	0.32	−0.02
> 5 years							
Adherence							
High^a^	**0.53**	0.31	0.01	0.10	0.12	0.33	0.30
Low							

^a^Reference category. Statistically significant ES are shown in bold

KIDSCREEN-27, KIDSCREEN-10 Index and Visual analogue scale (VAS)

Children and adolescents showed relatively few health problems in the EQ-5D-Y dimensions, although 33.6% reported having pain or discomfort and 26.1% reported having anxiety or depression; 50% of children scored the best possible health state on to the descriptive EQ-5D-Y system (data not shown).

Multivariate models of HRQOL are shown in Table 4. Age, single-parent families, adherence to treatment, and mental health were the influential factors on HRQOL. Statistically significant associated factors to the KIDSCREEN-10 were age (B coefficient = −0.93); single-parent families (B = −15.2); and mental health (B = −0.7). An interaction was found between age and type of family, so older ages showed less influence of single parent families on the KIDSCREEN-10 index of HRQOL (B = 1.02 for age and type of family interaction).

**Table 4:** Multiple linear regression models of the KIDSCREEN-27, KIDSCREEN-10 Index and Visual analogue Scale (VAS)

	Physical well- being *B (SE)*	Psychological well-being *B (SE)*	Parents relationship *B (SE)*	Peers *B (SE)*	School *B (SE)*	KIDSCREEN-10 B *(SE)*	VAS *B (SE)*
*Sociodemographics*							
Sex (boys)	-	-	-	-	−4.46 (1.52)^a^	-	-
Age	-	-	0.21 (0.08)^a^		-	−0.93 (0.25)^a^	-
-Type of family (monoparental)		-	-	-	-	−15.2 (4.05)^a^	-
*Clinical variables*-							
Adherence (high)	4.34 (1.48)^a^		-	-	-	-	-
Mental health (SDQ)	-	−0.96 (0.13)^a^	−0.46 (0.12)^a^	−0.71 (0.13)^a^	−0.63 (0.14)^a^	−0.7 (0.11)^a^	−0.62 (0.22)^a^
*Interaction terms*							
Age by type of family	-					1.02 (0.26)^a^	
*Adjusted R* ^*2*^	*0.15*	*0.29*	*0.12*	*0.17*	*0.16*	*0.30*	*0.08*

Reference category: girls; biparental family; adherence: low. Models are adjusted for the rest of variables in the Table and also level of education, origin, body mass index, time w/ diagnoses, and HBA1c

*VAS* Visual analogue scale, *B* beta coefficient, *SE* Standard error, *SDQ* Strengths and diffitulties questionnaire (total difficulties score)

^a^Statistically significant at 0.001 level according to Bonferroni correction

## Discussion

This study shows that children and adolescents with T1DM report similar HRQOL than the general population of the same age and gender, although slightly worse physical well-being than their peers. The study shows that the type of family, mental health and treatment adherence should be taken into account to improve HRQOL in these patients. It also shows the feasibility of assessing HRQOL via Internet, and its use as a daily clinical practice tool in a cohort of children, reinforcing the results of previous research [9, 10].

In general, HRQOL were similar than the general population, as it was shown in a previous systematic review [6]. In our study, younger patients had better HRQOL scores; and girls had lower HRQOL scores in virtually all dimensions, indicating greater vulnerability and similar results than in other studies [20, 21]. Thus, the study suggests the need to assess and monitor potential problems, especially in adolescence. Moreover, recent guidelines from ISPAD have recommended routine assessment of HRQOL with adequate tools [2]. Comparing our results with Spanish general population data Physical well-being dimension score was 0.5 standard deviation lower (worse) than the reference population, which represent a meaningful difference [22].

Other studies had already linked a worse HRQOL in children with specific characteristics such as those from single-parent families or families with disadvantaged socioeconomic status [23, 24]. The results of the present study reinforce this finding and the importance of knowing the social situation of the patient. One of the main findings indicates the need to provide special reinforcement in monoparental families at younger ages.

Evidence shows that better HRQOL is associated with better metabolic control although this relationship is modest [20, 21, 25, 26]. In our study it was also found an association between HRQOL and HbA1c; nevertheless, it is not possible to establish the directionality of association given the cross-sectional design of the analysis. Some studies suggest that the experience of having suffered severe hypoglycemia can affect HRQOL for fear of their recurrence [27]. In our study patients who had suffered significant hypoglycemia showed better scores in almost all dimensions of HRQOL, although these data are not valuable because of the small number of patients experiencing hypoglycemia. On the other hand, in general almost all patients were well controlled as it was reflected by the relatively few patients in the higher extreme of the curve distribution of HbA1c. This fact could be associated to the almost universal healthcare coverage and easy access to healthcare services and programmes in Spain, which facilitates disease control.

Adherence to treatment is very important to achieve a good metabolic control and it could be associated to HRQOL [21]. Our study shows similar results than a prospective study, with worse HRQOL in patients with a lower level of adherence [28]. Moreover, patients on pump therapy, requiring a very high level of adhesion, have been linked to improved quality of life [29, 30]. In our case we have not been able to analyze the characteristics of this treatment given the small number of patients.

Patients with poor mental health also show lower HRQOL scores in our study. This figures are similar than other studies in which diabetic patients have a high incidence of depression, anxiety and other psychological problems [24, 31, 32]. In this sense, the role of clinical psychologist could be important in order to reinforce those positive emotional and psychological aspects that are potentially modifiable in this group of patients [33]. Mental health and adherence were among the factors associated with HRQOL, therefore, are aspects to consider and evaluate regularly in diabetic patients, in addition to metabolic control.

The study has several limitations. Firstly, 35% of patients who did not enter the study could lead to a selection bias. Although there were no differences between these patients and those who participated in terms of age or years of evolution of the disease, we didn’t know the metabolic control of some of them. Perhaps some had higher levels of HbA1c and therefore would not participate. Secondly, patients were recruited from 5 different centers, so that personal characteristics, clinical factors and treatment received may vary the results. However, no significant differences were found in the multivariate models when entering the center or pediatrician (data not shown). Thirdly, the results, in part may be related to the HRQOL instruments used in each study. HRQOL studies in pediatric patients with T1DM have used different measurement instruments, some disease-specific, such as diabetes DISABKIDS module [34], or the Peds-QL [35].

One of the strengths of the study is the use of the KIDSCREEN and the EQ-5D-Y simultaneously, two generic HRQOL instruments for children. Thus, it has been possible to compare the HRQOL of this group of patients with the normative KIDSCREEN Spanish and European data and allowed us to estimate the impact comparing with the general population of the same age and sex. Moreover, it is currently not possible to calculate the Quality Adjusted Life Years (QALYs) or a similar indicator necessary to carry out a cost-effectiveness analysis due to the lack of developed preference-values in the Spanish population of this age group. The present study attempts to provide initial data to carry out such studies, which are scarce and necessary in childhood population. On the other hand, it was not included a specific HRQOL instrument in T1DM due to the lack of such instruments adapted into Spanish population of this age group. However both the KIDSCREEN and the EQ-5D-Y as generic instruments have been widely used in general healthy population as well as various health problems. Both instruments have demonstrated acceptable discrimination ability to study the impact of health problems on HRQOL. In addition, the availability of reference values has allowed us to use the European general population norms of the KIDSCREEN and compare with the sample with acceptable accuracy and easy interpretation. Finally, it has not been possible to include a standardized adherence measure given the lack of instruments adapted in Spain. However the variables used have yielded an assessment of adherence acceptably. Future studies should incorporate valid and reliable measures of this factor with potential utility in the daily clinical management.

## Conclusions

In summary, HRQOL in children and adolescents with T1DM were similar than the general population of the same age and gender, with slightly lower physical well-being. We have demonstrated the feasibility to assess HRQOL through the use of new technologies such as the Internet, which could provide essential elements in routine visits in the pediatric and adolescent diabetic patients.

## Abbreviations

DKA: Diabetes ketoacidosis
HbA1c: Glycated haemoglobin
HRQOL: Health-related quality of life
MDI: Multiple daily injections
T1DM: Type 1 Diabetes Mellitus
